# Diseases of Respiratory Organs

**Published:** 1905-07-08

**Authors:** 


### DISEASES OF RESPIRATORY ORGANS.

H. MAXON KING[1] has an interesting paper on the duration of tuberculosis. He believes that many apparently recent attacks are a recrudescence of those which began many years before and have been arrested at intervals, and similarly that many apparent cures are arrests due to acquired immunity which may easily be lost again. The occurrence of the initial lesion is often obscure and unrecognised; the patient usually reacts quickly and often successfully, and may retain good health for years. The history of numerous patients shows that a long period has existed between the probable date of infection and the first clinical symptoms, and that this period has been one of good health. The first infection is attended with a feverish attack and other constitutional disturbance, transient albuminuria, and possibly a diazo reaction. The white cells are at first slightly increased and the red diminished. The physical signs, if any, rapidly disappear, and the patient remains quite well till a recrudescence takes place. Bovaird[2] gives a very clear account of the dispute as to human and bovine tuberculosis, and on the whole supports Koch's view. He shows that human tuberculosis can only be transmitted to cattle with difficulty, and conversely, bovine tuberculosis rarely gains a footing in the human body. He thinks that there is evidence to show that in neither cattle nor men is there much disease due to the foreign bacillus. An astonishing thing is the rarity of cases of tuberculosis in children which can be traced to contaminated milk, when it is known that so many cattle are infected. Again, there is a curious difference between the frequency of cases with primary intestinal lesions in Germany, America, and Great Britain. In the last-mentioned country they are remarkably common. Bovaird is clear that these differences are not due to diversity of interpretation, but to some local factors not taken into account. In various districts of Germany, too, there is no fixed relation between the frequency of tuberculosis in man and the number of diseased cattle in a given district. If anything there is less human disease where the cattle are frequently tuberculous and much milk is consumed. Kitasato points out that while tuberculosis is rife in Japan, the native cattle are exempt, though imported animals and cross-breeds suffer as in Europe. Hence the prevalence of human tuberculosis in former days was not due to bovine infection.

**Pleural Effusions.**—Godlee[3] shows in his Hunterian lecture that dulness from empyema with no gas does not shift at all with alteration of the patient's position, and that dulness from serous effusion shifts very little. In pyo-pneumothorax or sub-diaphragmatic conditions shifting does take place. S. West[4] points out that this immobility has long been recognised and is probably due to the collapse of lung immediately adjacent to the fluid while the rest of the lung is more distended. This change in the lung is not easily altered. It is curious to find in opposition to this evidence that Barjon and Courmont,[5] from examination by the *x*-rays, contend that effusions are freely movable with the position of the patient, but they acknowledge that the ordinary view is correct that the upper limit is usually marked by a curve with its highest point in the axillary line. G. A. Gibson[6] considers that an excess of lymphocytes in a pleural effusion is indicative of the stage and extent of reaction present, rather than of a tubercular origin. In a definite tubercular hydropneumothorax he has found an excess of polynuclear cells, and also in pneumococcal cases, while in malignant disease of the pleura there is an excess of eosinophiles and myelocytes, and in some rheumatic cases an excess of lymphocytes. He found that in 118 cases of pleurisy 29·7 per cent. were tuberculous, and 22·8 per cent. were pneumococcal, while in 28·8 per cent. no cause could be discovered. He does not believe that all the last group were tuberculous, and thinks that in many cases of tuberculosis the disease is the result of a second and distinct infection, and that we cannot even consider that the structural damage caused by pleurisy conduced to it. However, a large lymphocytosis in the fluid at an early stage, where it is not produced by rheumatism, and the absence of leucocytosis in the blood are characteristic of tuberculous pleurisy. Schiller[7] discusses Bulau's method of treatment of an empyema by continued aspiratory drainage. It has been claimed that the method prevents pneumothorax, and enables us to do without both an anæsthetic and a large wound. However, with restless patients it is impossible, the process is a long one, and the tube is apt to get blocked. Still the method may be employed in double empyema, or where there is great exhaustion or dyspnœa. In tuberculous cases, if the pus is sterile, puncture and the injection of iodoform may be used. Jehle[8] records a remarkable complication of empyema. A fistulous opening from the œsophagus into the pleural cavity was formed, probably by the breaking down of a gland, and food matters found their way in while the cavity was being drained. The fistula, however, healed, and the empyema was eventually cured. Courmont and Nicolas[9] have studied the urine during pleurisy. They find that during the febrile stage, when the effusion is increasing, the urine is diminished and the chlorides in it are low, but when absorption is taking place, the state of matters is reversed, and albuminuria is frequent. They think that the chlorides are at first fixed and employed to form antidotal bodies, and that we should therefore at the onset of the attack give chlorides freely, but that these should be cut off when the fluid is abundant, or the urine shows an excess. Chauffard and Bodin, however,[10] found that chlorides given in the acute stage are harmful, and advise a milk diet at first. Chlorides not only increase the amount of effusion, but produce mental excitement and may even lead to venous thrombosis.

**Pneumonia.**—Manges[11] remarks that this is an acute general disease, that the lung affection is only one of the local lesions, that the blood nearly always contains the cocci, and that the lung is much the same after the crisis as before it. Since the advent of influenza the lung affection is more often irregular. Wells has shown that the death rate in 465,000 cases was 20 per cent. for all ages, and Fränkel gives the rate for large cities as between 20 and 35 per cent. It is important to prevent distension of the stomach and bowels, even aerated waters are harmful. The blood-pressure is low, indicating often a vasomotor paresis. Caffeine, large doses of strychnine, hypodermics of ergot, saline injections, and adrenalin are recommended. Digitalis may be of use when the pulse-rate increases and becomes irregular and small. If moist rales appear in the unaffected lung dry cupping and atropine should at once be used. In young, robust adults venesection is sometimes needed. Cold baths are harmful, but icebags and sponging may be helpful. For the pleural pain minute doses of morphine, or the Paquelin cautery are best. Sleep by day and by night must be sought by every means, especially by a selection of the newer hypnotics. Oxygen may be of some little use in tiding over attacks of dyspnœa and cyanosis, but generally it can do little. Ewart[12] agrees in pointing out that the disease is a general infection, and that the lungs may entirely escape. When they are attacked it is only a portion of them which suffers, possibly the area supplied by a single vessel. The fibrin effused in this area is a culture ground in which the diplococci multiply and secrete their poison. While he deprecates merely expectant treatment, he grants that no definite method has universal assent, many specifics are recommended, each by a few supporters. He urges that treatment should be early and active. When the first rigor occurs a hot stimulant may be given with bromide to ensure rest, to be followed by calomel and senna. To relieve the heart he would give oxygen early as a stimulant. Leeches, if applied early, do more than relieve pain, though they do this better than any other means. Venesection is only of exceptional use. Diaphoresis should be encouraged by ammonium citrate and sweet nitre. Gin gives the advantages of alcohol, and of a diuretic. Citrates and iodides check coagulation; but carbonates should be avoided because of the danger of gastric distension. Upon iodide of potassium in frequent doses throughout the disease he puts great value. As to diet, he prefers to give at first little except whey, water, and alcohol, and later eggs with raw meat juice, maltine, predigested foods, and milk in tea or cocoa. Strychnine and quinine are added to the previous medicines. Attention must be given to sleep and the support of the heart, but digitalis appears to be rarely included in his list. The question of the withdrawal of chlorides from the diet is not capable of a definite decision.

[1] Med. Rec., Jan. 7. [2] Ibid., Feb. 25. [3] Lancet, Feb. 28. [4] Ibid., March 18. [5] Edin. Med. Jour., Dec. [6] Brit. Med. Jour., Jan. 7. [7] Edin. Med. Jour., Dec. [8] Lancet, Oct. 18. [9] Edin. Med. Jour., Dec. [10] Amer. Jour. Med. Sci., Jan. [11] Med. Rec., Dec. 10. [12] Lancet, Jan. 21.

